# Valorization of CO_2_ through the Synthesis of Cyclic Carbonates Catalyzed by ZIFs

**DOI:** 10.3390/molecules27227791

**Published:** 2022-11-12

**Authors:** José J. Delgado-Marín, Iris Martín-García, David Villalgordo-Hernández, Francisco Alonso, Enrique V. Ramos-Fernández, Javier Narciso

**Affiliations:** 1 Instituto de Materiales, Departamento de Química Inorgánica, Facultad de Ciencias, Universidad de Alicante, Apdo. 99, 03080 Alicante, Spain; 2 Instituto de Síntesis Orgánica, Departamento de Química Orgánica, Facultad de Ciencias, Universidad de Alicante, Apdo. 99, 03080 Alicante, Spain; 3 Instituto de Investigación Sanitaria Biomédica de Alicante (ISABIAL), 03690 Alicante, Spain

**Keywords:** carbon dioxide, cyclic carbonates, epichlorohydrin, epoxides, heterogeneous catalysis, ZIFs

## Abstract

One way to exploit CO_2_ is to use it as a feedstock for the production of cyclic carbonates via its reaction with organic epoxides. As far as we know, there is still no heterogeneous catalyst that accelerates the reaction in a selective, efficient and industrially usable way. Cobalt and zinc-based zeolitic imidazole frameworks (ZIFs) have been explored as heterogeneous catalysts for this reaction. In particular, we have prepared ZIF-8 and ZIF-67 catalysts, which have been modified by partial replacement of 2-methylimidazole by 1,2,4-triazole, in order to introduce uncoordinated nitrogen groups with the metal. The catalysts have shown very good catalytic performance, within the best of the heterogeneous catalysts tested in the cycloaddition of CO_2_ with epichlorohydrin. The catalytic activity is due ultimately to defects on the outer surface of the crystal, and varies in the order of ZIF-67-m > ZIF-67 > ZiF-8-m = ZIF-8. Notably, reactions take place under mild reaction conditions and without the use of co-catalysts.

## 1. Introduction

Nowadays, one of the main environmental challenges is the reduction of the greenhouse effect, caused by a reduction in the generation of certain gases (especially CO_2_). The easiest way to reach this goal is to decrease the use of fossil fuels by substitution with renewable energies, the use of more efficient engines, and hybrid or electric cars. Norway is a paradigmatic country where electricity is practically obtained from renewable energies. Different strategies have been proposed for CO_2_ mitigation, perhaps the most effective so far being to utilize CO_2_ for the preparation of cyclic carbonates (and also at an industrial level) through the cycloaddition of CO_2_ with epoxides [1]. Obviously, the production of cyclic carbonates and polycarbonates is very low compared to the global release of CO_2_ to the atmosphere, but it is an avenue for sequestering CO_2_ that should be explored (since apart from the current uses of polycarbonates, these could replace some of the plastics we use today). The two most widely used polycarbonates are based on ethylene oxide and propylene oxide, both of which are obtained from the petroleum industry (which is not a renewable source). However, efforts are being made to find other routes for the production of these precursors, such as those based on bioalcohols derived from biomass [2]. Limonene oxide has also been described as an alternative epoxide, whose precursor monoterpene is obtained from the citrus peel used in the production of juices [3]. Perhaps the most interesting alternative is the use of glycerol as a precursor of glycidyl alcohol, which is a by-product in the synthesis of biodiesel. However, to date, the results obtained from this approach have not been very promising [4,5,6]. However, epichlorohydrin can be obtained from glycerol, which is one of the most widely used epoxides to synthesize cyclic carbonates, at least at an academic level [7,8,9,10]. These types of reactions are catalyzed both under homogeneous and heterogeneous conditions. In homogeneous catalysis, the most used catalysts are, for example, quaternary ammonium salt halides (tetrabutylammonium halides, TBAX) and imidazolium halides (with regards to Lewis-base homogeneous catalysis) [11,12]. In the case of acid catalysis, metal porphyrins have been widely used [13,14]. The main problem of homogeneous catalysis is the need to separate the catalyst from the products, something that is usually carried out by distillation, which is a large energy-consumer purification technique. In heterogeneous catalysis, one of the most used routes is the immobilization of an active phase (Lewis base) on a support, such as MCM41 [15,16] or SBA-15 [17,18]. Concerning acidic sites, Cr-MIL-101 [19,20] is one of the most efficient catalysts, where the metal loses two coordination water molecules when it is activated, forming what is called the open metal site (Lewis acid character). However, it is known that both ZIF-8 and ZIF-67 are also catalytically active [21,22], which in principle should not be. These three latter catalysts are MOFs (metal organic frameworks [23,24]), which are basically metallic nodes or clusters linked through organic ligands, which generate a three-dimensional structure with a high porosity that can exceed 4000 m^2^/g in some cases. The advantage of ZIFs (zeolitic imidazolate framework), especially ZIF-8 and ZIF-67, over most MOFs is their high thermal (>400 °C, air) and water vapor stability. In addition, at present, there are already synthetic routes to MOFs that are found within the so-called ‘Green Chemistry’, since their synthesis is carried out in an aqueous medium at moderate pH. ZIF-8 and ZIF-67 are isostructural, the difference between them is that Zn^2+^ is the cation in the first case and Co^2+^ in the second case. Unlike Cr-MIL-101, ZIF-8 and ZIF-67 are catalytically active since ZIFs have some defects (that is to say, the metal node can have coordinatively unsaturated sites displaying Lewis acid behavior) [25]. As the coordination is carried out with the nitrogen atoms of 2-methylimidazole (linker), this implies that a possible adjacent basic center could be generated. One of the possible strategies to improve the efficiency of these ZIFs could be to search for synthetic routes that promote structural defects, or as a more elegant approach, the partial substitution of 2-methylimidazole for a triazole to significantly increase the number of basic sites.

**Scheme 1:**
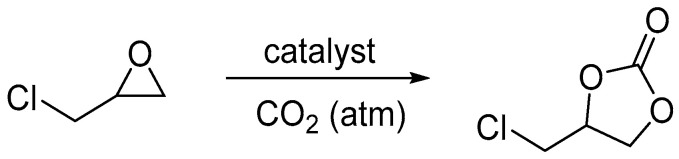
Reaction of fixation of CO_2_ in epichlorohydrin to give 3-chloropropene carbonate.

In the present investigation, four catalysts have been synthesized (ZIF-8, ZIF-67, ZIF-8-m and ZIF-67-m) where ‘m’ indicates that there has been a partial substitution of 2-methylimidazole for a 1,2,4-triazole, maintaining the ZIF structure. Their catalytic activity has been compared in the reaction of CO_2_ with epichlorohydrin to give the corresponding cyclic carbonate (Scheme 1). The effect of the temperature, pressure, reaction time, and the incorporation of TBAI as a cocatalyst have also been studied.

## 2. Results and Discussion

**Figure 1:**
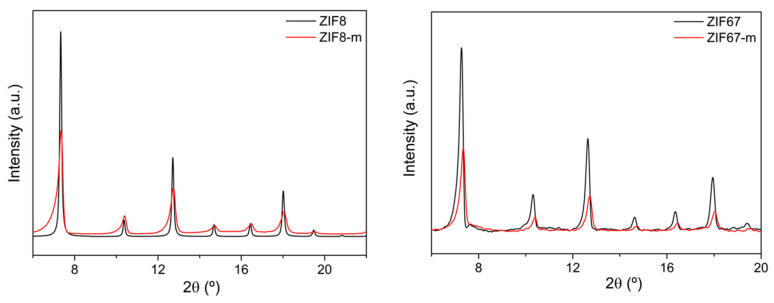
X-ray diffraction patterns of the four catalysts.

Four catalysts have been prepared, which are in principle isostructural, something that can be clearly seen in the diffractograms shown in Figure 1. The diffraction patterns of ZIF-8 and ZIF-67 are exactly the same as those previously published. However, the catalysts modified with 1,2,4-triazole present certain differences, especially ZIF-67, where the peaks appear shifted and there is a variation in the relative intensity. This indicates that the exchange of the ligands produces a change in the unit cell and a small loss of crystallinity. It is important to remember that Zubieta and co-workers have carried out an exhaustive study on the synthesis of MOFs [26,27] with bimetallic cations and 1,2,4-triazole, and in no case they obtained an isostructural compound with ZIF-8. Thus, a topological change could be induced if the degree of exchange is very high. In the case of ZIF-8, a shift to larger angles is mainly observed, indicating that the structure is the same but, as expected, the lattice parameters change since the ligand is slightly smaller. 

**Figure 2:**
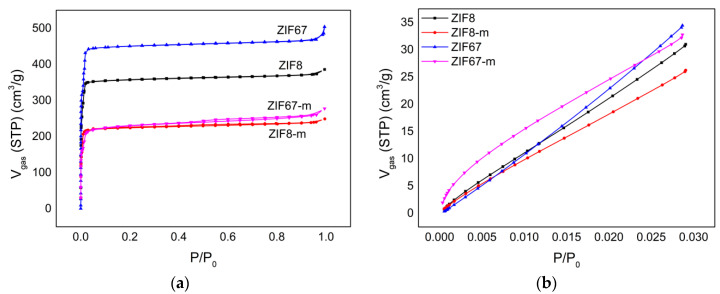
Adsorption isotherm of the four catalysts. (**a**) N_2_ at –196 °C; (**b**) CO_2_ at 0 °C.

Figure 2 and Table 1 show the results of adsorption of the four catalysts. The surface area does experience a great change, especially in ZIF-67, as the observed isotherm is type I in all cases, albeit ZIF-67-m seems to present a slight mesoporosity. But the most remarkable thing is that the typical step at low relative pressures appears for all of these catalysts, which is comparable to the fingerprint of these materials. In principle, it would be expected that with such a drastic reduction in the area of ZIF-8-m and ZIF-67-m (60 and 50% respectively), the adsorption capacity of the catalysts should be less efficient, even though the incorporation an extra N atom into the structure should favor the adsorption of CO_2_. The CO_2_ adsorption isotherms are very interesting: it can be seen that there is no difference in the type of adsorption for ZIF-8 and modified ZIF-8, both cases being linear, but the maximum adsorption capacity only decreases a 10%, while the decrease in the surface area is a 40%.

Much more notable is the case of ZIF-67, where the reduction in the CO_2_ adsorption capacity is only 10%, while that of the surface area is almost 60%, as mentioned above, and now it is no longer linear but rather shows a greater affinity for CO_2_ than in the case of the ZIF-67. The aforementioned shows that, in this case, there must be a change of greater scope than just the replacement of the linkers. 

**Figure 3:**
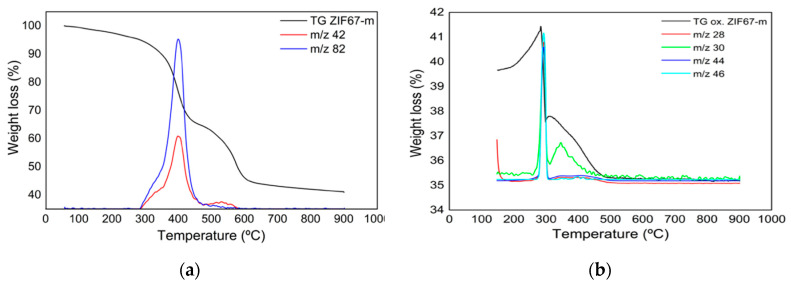
TGA-MS of sample ZIF-67-m. (**a**) In Ar, where *m/z* 42 is related with the 1,2,4-triazole and *m/z* 82 is related with 2-methylimidazole; (**b**) in 4:1 Ar:O_2_, where *m/z* 28 is CO, *m/z* 30 is NO, *m/z* 44 is CO_2_, and *m/z* 46 is NO_2_.

A semi-quantitative determination of the degree of exchange can be carried out with the TGA-MS technique. The protocol used was as follows. First, the catalyst was heated to 900 °C in an Ar atmosphere. Then, the sample was cooled to 50 °C and the process was repeated, but this time in a 4:1 Ar/O_2_ mixture. Air was not used to avoid overlapping of N_2_ signals with those of CO. In the first stage, a carbon material with a high nitrogen content and the metal [11] was obtained; in the second stage, the carbon phase was eliminated and the metal remained either as ZnO or as Co_3_O_4_, allowing us to approximately quantify how much linker was exchanged. In the case of ZIF-67 (Figure A2) and ZIF-8, a single very well-defined weight loss was observed around 600 °C, and the final mass of oxide allowed us to identify that everything was correct (99% in both cases). This small difference can be attributed to an error in the quantification, such as the presence in the sample of a small amount of solvent, some defects, etc. Figure 3 shows the TGA-MS of the modified catalysts. First of all, it should be noted that the TGA curves are more complex, basically observing two zones, one at low temperature (300–400 °C) and another more irregular above 600 °C. This indicates that the material is much less stable. In addition, it is observed by MS that both linkers come out mainly at much lower temperatures than that of the unmodified ZIFs, and that the weight loss is greater in the first stage (Ar), followed by a second weight loss that can be attributed to the formation of a carbon material. 

In the second stage, we observed the combustion of the carbon material and the oxidation of the metal. It is also notable that the C/N ratio decreases quite a bit based on the ratio of the CO_2_/NO_2_ areas, indicating that the formed carbon material contains a higher N content, what is very interesting. Now the content of ZnO and Co_3_O_4_ is different. If we assume that no other phase is being formed, we can deduce that the degree of exchange is 25% in the case of ZIF-8 and 30% in the case of ZIF-67. 

**Figure 4:**
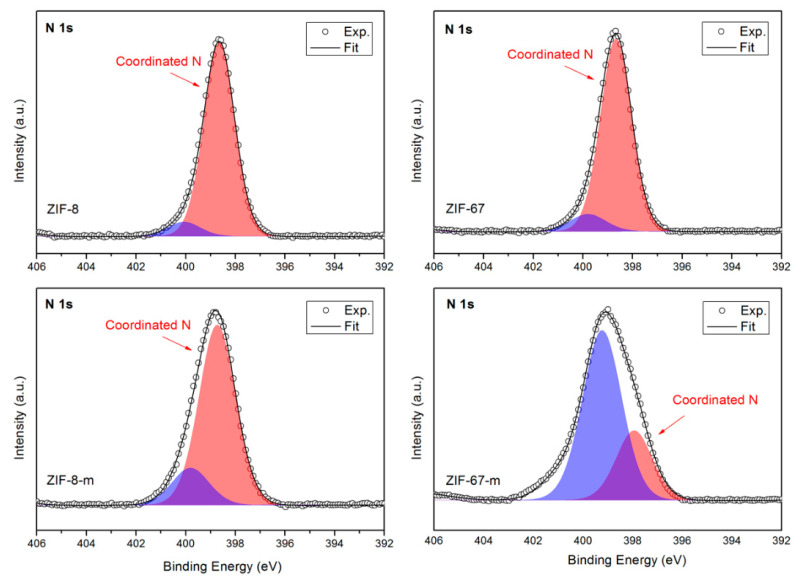
XPS spectra of the four catalysts.

Figure 4 shows the XPS spectrum of the four catalysts developed; only the N signal is shown since, in principle, it is the most relevant, while the metal spectra are shown in the Appendix A (Figure A3). Before analyzing the spectra in detail, it must be noted that the nitrogen-to-metal ratio increases with the modified catalysts (as expected). In principle, the N atom not bound to N should give a different signal from that bound. It is clearly observed that there is a use of the non-coordinated N signal. The presence of the said N in the unchanged catalyst is indicative that there are defects in the coordination. In the case of the ZIF-67-m, the largest signal may be indicative that there are a greater number of defects or that a new phase is being formed where the coordination may be changing.

**Figure 5:**
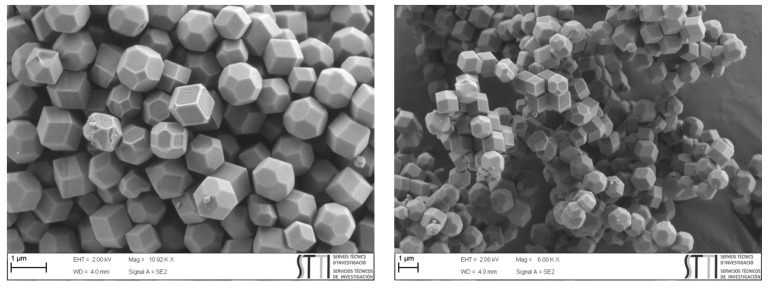
Secondary electron micrographs of ZIF-67.

Figure 5 shows two electron micrographs of ZIF-67, where it can be seen that it has a very regular distribution where crystals with a truncated octahedron shape predominate, with an average size of about 400 nm. In the exchange process, no modification has been observed in the microstructure or in the surface of the faces of the monocrystals.

**Figure 6:**
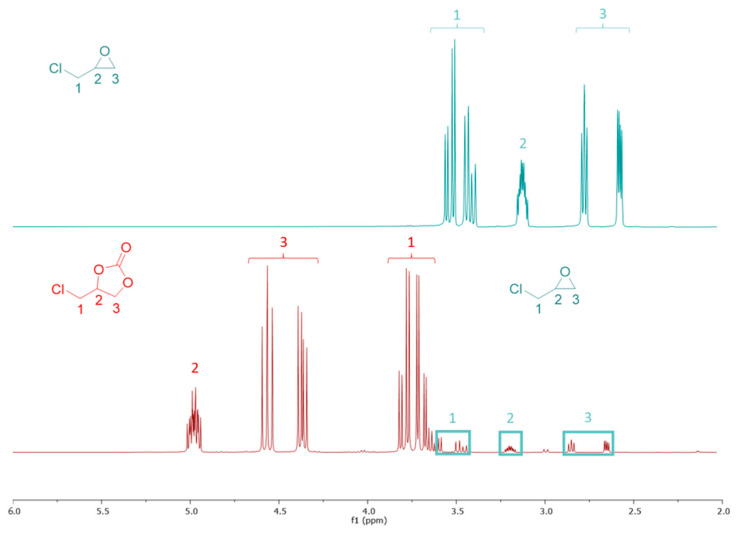
^1^H-NMR spectra of epichlorohydrin (blue) and a reaction crude (red).

**Figure A1:**
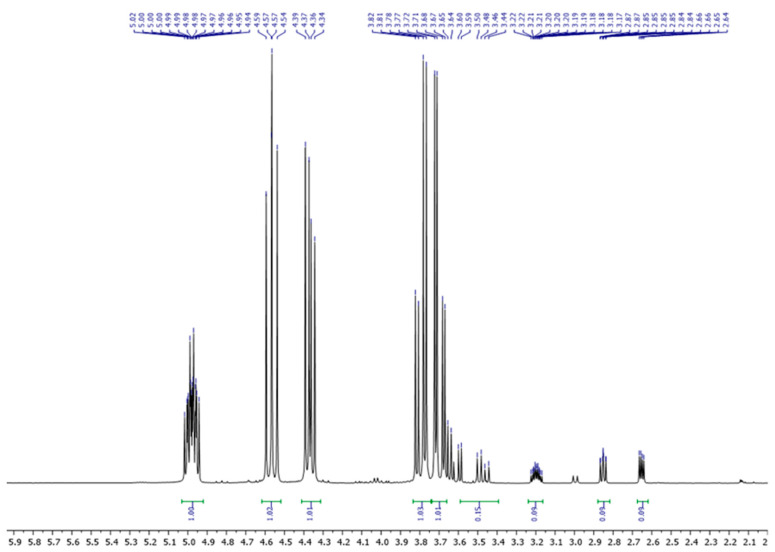
^1^H-NMR spectra of the reaction crude.

**Figure A2:**
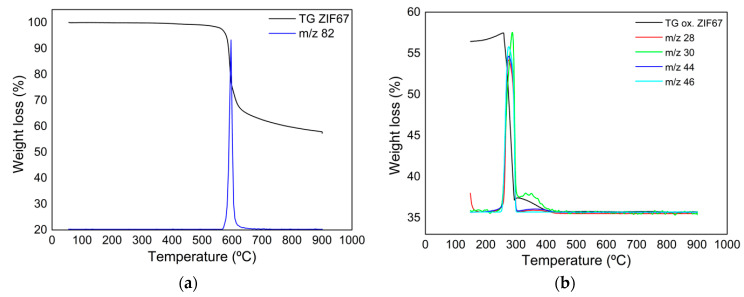
TGA-MS of sample ZIF-67. (**a**) In Ar, where *m/z* 82 is related with the 2-methylimidazole; (**b**) in 4:1 Ar:O_2_, where *m/z* 28 is related to CO, *m/z* 30 is NO, *m/z* 44 is CO_2_, and *m/z* is 46 NO_2_.

**Figure A3:**
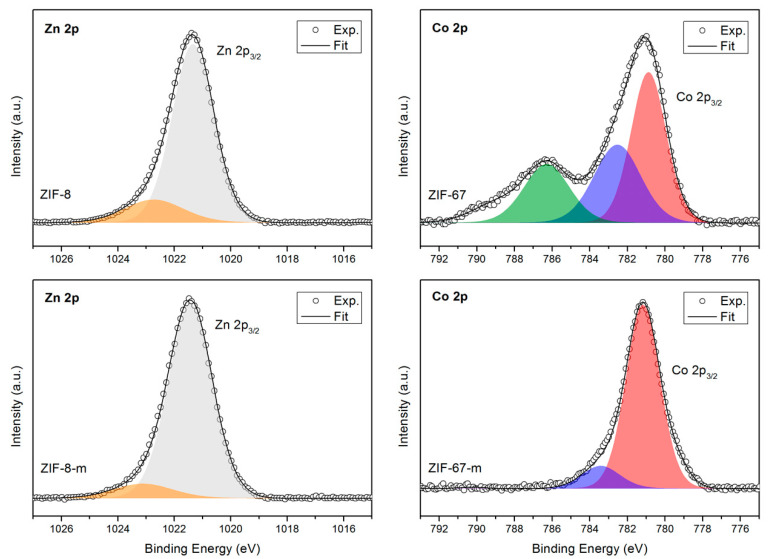
XPS of the four catalysts.

In order to obtain the conversion of epichlorohydrin to 3-chloropropene carbonate, the reaction products were analyzed by ^1^H NMR. Figure A1 shows the ^1^H-NMR spectra in great detail, where it can be clearly seen that the only compounds appearing in the reaction crude are the starting epichlorohydrin and its derived carbonate. Figure 6 shows the ^1^H NMR spectrum of epichlorohydrin and a typical spectrum of one of the reaction-crude aliquots after filtration. The fact that the spectra are very clean makes the quantification of both compounds simple. Given that no other products have been detected in any of the experiments carried out, the selectivity achieved is 100%.

**Figure 7:**
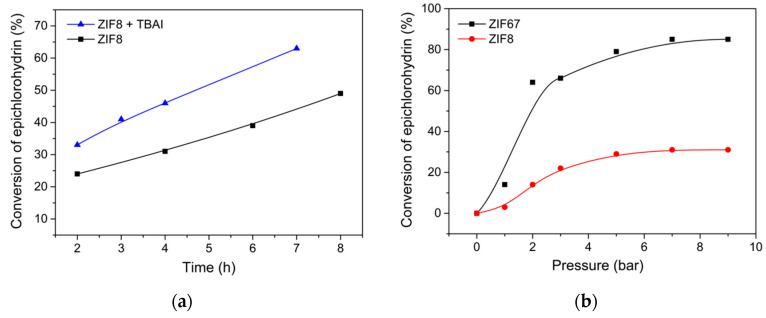
(**a**) Study of the effect of co-catalyst in the reaction of CO_2_ with epichlorohydrin using ZIF-8 as catalyst. Conditions: 97.8 mol.% of epichlorohydrin, 0.6 mol.% of ZIF-8, 1.4 mol.% of TBAI, 60 °C, 7 bar CO_2_. (**b**) Effect of the pressure in the catalytic reaction of cycloaddition of CO_2_ with two different catalysts (ZIF-8 and ZIF-67). Conditions: 99.4 mol.% of epichlorohydrin, 0.6 mol.% of ZIF catalysts, 120 °C, 4 h. The conversion of epichlorohydrin was based on ^1^H-NMR results (3-chloropropene carbonate was the only product).

As indicated in the introduction, tetrabutylammonium halide salts are good homogeneous catalysts for the title reaction and, therefore, they can be also used as cocatalysts [28]. First of all, its effect and its obligatory presence in the reaction have been verified. As it can be seen in Figure 7a, the presence of TBAI improves the catalytic results of ZIF-8, but ZIF-8 itself already shows good catalytic behavior; that is why the use of TBAI was not considered necessary.

The three main variables studied in the reaction are CO_2_ pressure, time, and temperature. First, the effect of pressure on the reaction was analyzed. The data obtained are shown in Figure 7b. As expected, there is a rapid increase in the conversion with pressure, which becomes steady from 7 bars. Some authors [29] obtained similar results, and commented that the conversion decreased drastically if the pressure was greater than 20 bars; we believe that this behavior maybe a consequence of the collapse of the MOF structure. 

The effect of the temperature is shown for ZIF-8-m, where a linear increase in conversion with temperature can be seen, although it is usually exponential, which indicates that there are other factors controlling the reaction rate. 

**Figure 8:**
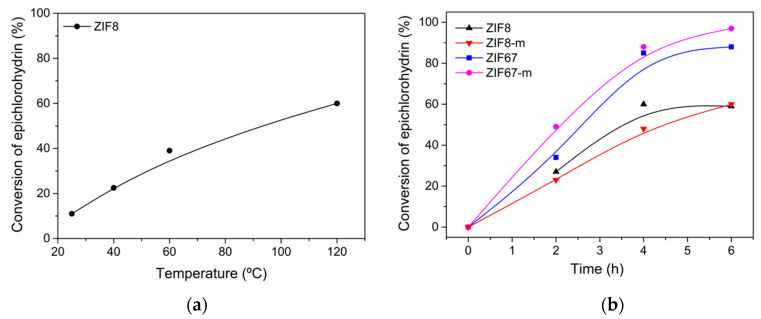
(**a**) Effect of the temperature in the catalytic cycloaddition of CO_2_ with epichlorohydrin using ZIF-8. Conditions: 99.4 mol.% of epichlorohydrin, 0.6 mol.% of ZIF catalysts, 7 bar CO_2_, 6 h. (**b**) Catalytic reaction of cycloaddition of CO_2_ with four different catalysts (ZIF-8, ZIF-8-m, ZIF-67 and ZIF-67-m). Conditions: 99.4 mol.% of epichlorohydrin, 0.6 mol.% of ZIF catalysts, 7 bar CO_2_, 120 °C. The conversion of epichlorohydrin was based on ^1^H-NMR results (3-chloropropene carbonate was the only product).

Once the pressure and temperature were established, we analyzed the effect of time (Figure 8b), where it is clearly observed that ZIF-67 is a better catalyst than ZIF-8, and that the modified catalyst in the case of ZIF-67 does experience an improvement, while in the case of the ZIF-8 it is not appreciable. Although, apparently, the improvement is not substantial, in reality it is very remarkable since, if we take into account the decrease in the volume of the micropores, the activity of the ZIF-67-m catalyst per unit area is 268% more effective. This actually occurs since the extra N atom in the triazole is even more effective than the other two nitrogen atoms coordinated to the metal, according to the mechanism we have proposed.

**Figure 9:**
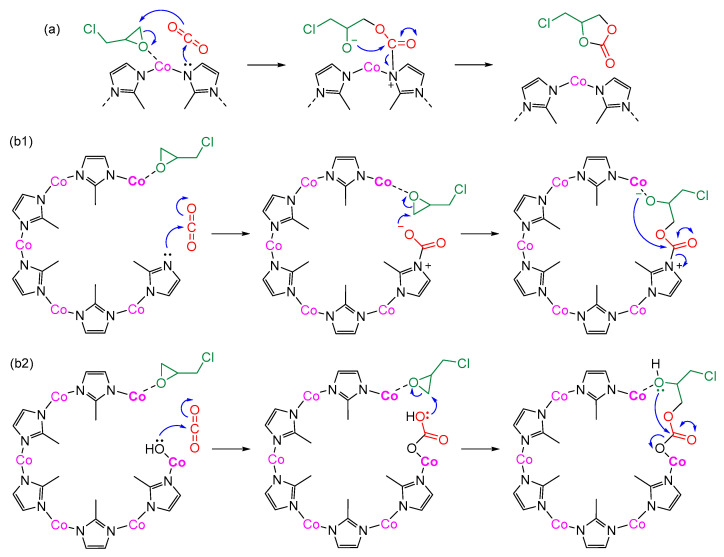
(**a**) Classical reaction mechanism for the cycloaddition of epichlorohydrin and CO_2_ catalyzed by ZIF-67. (**b**) Proposed reaction mechanism with ZIF-67 involving structural defects. (**b1**) CO_2_ activation by a partially coordinated 2-methylimidazole unit. (**b2**) CO_2_ activation by a superficial hydroxyl group.

Concerning the reaction mechanism, different reports describe how ZIF-67 and ZIF-8 materials can activate the epoxide and CO_2_ towards the cycloaddition reaction (Figure 9a) [7,21]. It is generally assumed that the acidic and basic sites on the external surface and/or structural defects can account for the catalysis [10,30]. The atmospheric moisture and CO_2_ can contribute to the formation of superficial OH and NH groups, together with hydrogenocarbonates [31]. Although these groups might activate the epoxide by hydrogen bonding [10], the presence of low-coordinated Co(II,III) ions is expected to exert a stronger activating effect on the epoxide as Lewis acids. These kind of defects can leave free N atoms in the 2-imidazolate ligands, which have been suggested to activate CO_2_ by nucleophilic attack on the carbon atom. Nucleophilic attack of the resulting species on the Co-activated epoxide, followed by intramolecular cyclization, would give the corresponding cyclic carbonate (Figure 9(b1)). In our opinion, the possibility of superficial OH activating CO_2_ must not be ruled out [10] (Figure 9(b2)). Indeed, coordination of the Lewis-acidic Co ion to the pyrrolic nitrogen of 2-methylimidazole could have an electron withdrawing effect on the heterocyclic ring, decreasing its nucleophilic character and, consequently, the activation power of the free N atom on CO_2_, unless the 2-methyl and the metal-ligand backbonding could compensate for this effect [32]. An additional decrease in the reactivity of the imidazolate unit due to the steric effect of the 2-methyl group must not be disregarded. The possibility of a coordinated N atom to the metal (N-Co) activating CO_2_ must be totally discarded due to its depleted nucleophilic character and steric constraint.

**Figure 10:**
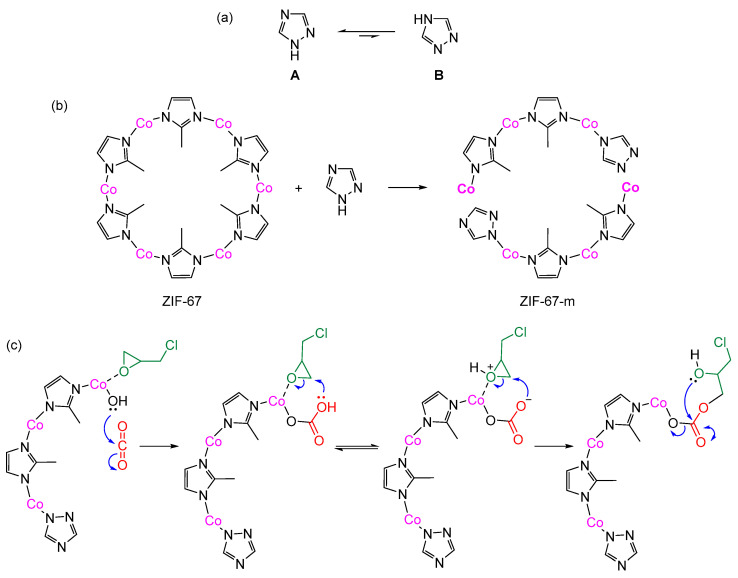
(**a**) Tautomeric forms of 1,2,4-triazole. (**b**) Proposed structural modification of ZIF-67 with 1,2,4-triazole. (**c**) Proposed reaction mechanism for the ZIF-67-m-catalyzed cycloaddition of epichlorohydrin and CO_2_.

As regards 1,2,4-triazole, different theoretical and experimental studies support that the tautomeric form A predominates or is the exclusive one (Figure 10a) [33,34]. It shows a decreased basicity (pKa 10.3) with respect to that of 2-methylimidazole (pKa 14.4) and it is deactivated against electrophilic attack, thus resembling the electronic character of pyridine [35]. We believe that the reduced basicity of 1,2,4-triazole favors the formation of more defects when introduced into the structure of the ZIF (Figure 10b), increasing the proportion of low-coordinated Co(II,III) ions and improving the performance of the catalyst (Figure 10b). The lack of nucleophilic character makes the activation of CO_2_ by a 1,2,4-triazolate unit very improbable. However, an activation similar to that in Figure 9b could take place, where two 2-methylimidazolate units could participate, one activating the epoxide and the other one CO_2_ (the latter through Co-OH species). However, if two vacants are available on Co, activation of both CO_2_ and the epoxide on the same site would be also feasible (Figure 10c). This proposal is particularly interesting if we take into account that the same Co site would put into spatial proximity both components, the epoxide and CO_2_.

## 3. Materials and Methods

The synthesis of catalysts followed a procedure as reported elsewhere [36,37], in which 144 mmol of 2-methylimidazole were dissolved in 50 mL of deionized water and 12 mmol of cobalt(II) or zinc(II) acetate were dissolved in 25 mL of deionized water. Then, both solutions were blended and stirred vigorously during 5 min and kept during 72 h at room temperature; any of the MOF precipitates was separated by centrifugation and washed thrice with MeOH. Finally, the MOF powder was dried for 24 h at 60 °C in a conventional oven. All chemicals have been provided by Aldrich (analytical grade) and has been used without further purification. Once the MOF was prepared, the partial transformation of ZIF-8 or ZIF-67 into ZIF-8-m or ZIF-67-m has been accomplished following the next procedure: 200 mg of ZIF were blended with 200 mg of 1,2,4-triazole; the blend was placed in a vial, the vial was then flushed with N_2_ and sealed with a silicone cap. The sealed vial was placed in a pre-heated oven at 130 °C and kept in the oven for 8 h. After that, the vial was broken and a solid was obtained. The solid was composed of ZIF-8-m or ZIF-67-m, together with some impurities (i.e., unreacted linker). Purification was performed by Soxhlet extraction, using acetone as a solvent for 24 h.

The porosity of the samples was characterized by means of nitrogen adsorption-desorption isotherms and CO_2_ adsorption isotherms. Samples were outgassed at 150 °C for 4 h prior to the adsorption measurements. The nitrogen adsorption-desorption isotherms were measured at –196 °C in a Quadrawin (Quantachrome) device. S_BET_ was determined from the N_2_ adsorption branch. In all cases, the number of points used to apply the BET equation was higher than 5, and the value of c was always positive. The CO_2_ adsorption isotherms were measured at 0 °C in the same device. V_micro_ was estimated by the Dubinin-Raduskevich method, with the objective to determine whether any diffusional restrictions in the adsorption took place. 

Crystallographic phases were identified by powder X-ray diffraction (PXRD), recorded on a Brucker D8-Advanced diffractometer with a Goebel mirror and a Kristalloflex K 760-80F X-ray generation system (K-alfa, λ = 1.54 Å), fitted with a Cu cathode and a Ni filter. Spectra were registered between 3° and 40° with a step of 0.05° and a step time of 3 s.

XPS measurements were acquired in a VG-Microtech Multilab device (VG-Microtech, UK) with a Mg-Kα (Hv: 1253.6 eV) radiation source, pass energy of 50 eV and background pressure of 5 × 10^−7^ Pa. A careful deconvolution of the spectra was made; the areas of the peaks were estimated by calculating the integral of each peak after subtracting a Shirley background and fitting the experimental peak to a combination of Lorentzian/Gaussian lines of 30/70 proportions.

The morphology of the samples was studied by Field Emission Scanning Electron Microscopy with X-ray microanalysis (FESEM–EDS) (ZEISS-Merlin VP Compact, BRUKER-Quantax 400) in both, Backscattered Electron (BSE) and Secondary Electron (SE) modes. 

The characterization of the samples by means of TG-DTA-MS was carried out in a TGA/SDTA851e/LF/1600 apparatus from Mettler Toledo, equipped with the Thermostar GSD301T Pfeiffer mass spectrometer. The TG experiments were carried out in the dynamic atmosphere of Ar (100 cm^3^/min), with a heating rate of 10 °C/min, while scanning masses up to 200 amu.

Reactions were carried out in a stainless-steel HPM-Vivor autoclave (Premex Solutions Gmbh); volume = 60 mL; P_max_ = 300 bar; T_max_ = 200 °C. The epoxide (epichlorohydrin, 18.0 mmol), catalyst (0.108 mmol) and, in some experiments, quaternary ammonium salt (TBAI, 0.288 mmol) were added to the reactor. The reactor was purged three times followed by the introduction of CO_2_ gas (1–9 bar,). The reaction mixture was stirred at 25–120 °C for 1–12 h. The resulting mixture was cooled to room temperature and collected in a vial to do the analysis by ^1^H-NMR.

^1^H-NMR spectra were recorded on Bruker Avance 300 and 400 spectrometers (300 and 400 MHz, respectively); chemical shifts are shown in parts per million (δ) and coupling constants (*J*) in Hertz (Hz). The ^1^H-NMR conversions were determined from the reaction crudes using mesitylene as an internal standard or the signal of the limiting reagent. Samples (5–10 mg) were taken from the reaction crude after filtration through a pad containing layers of Celite and MgSO_4_, and were diluted with CDCl_3_ (0.5 mL) as solvent. The ^1^H-NMR spectra are shown in the Appendix A (Figure A1). 

## 4. Conclusions

Four catalysts have been synthesized with the same structure based on ZIF-8, where the metal Zn has been changed into Co to generate ZIF-67. The linker in ZIF-8 and ZIF-67, 2-methylimidazole, has been partially exchanged for 1,2,4-triazole, giving rise to ZIF-8-m and ZIF-67-m. The degree of exchange is approximately 25% for both ZIFs. The exchanged catalysts (ZIF-8-m, ZIF-67-m) show a BET area decrease between 40–60% while the CO_2_ adsorption capacity practically does not vary. An important fact is that Co^2+^ is partially oxidized to Co^3+^.

In the catalytic process, it has been found that the four catalysts are very active in obtaining the cyclic carbonate derived from CO_2_ and epichlorohydrin. It is noteworthy that mild conditions have been applied and the use of cocatalysts has not been necessary. The catalytic activity varies in the following order: ZIF-67-m > ZIF-67 > ZIF-8-m = ZIF-8. The higher catalytic activity of the modified catalysts has been mechanistically rationalized in terms of structural defects.

## Figures and Tables

**Table 1:** Textural properties of the four catalysts obtained from N_2_ adsorption.

Sample	BET (m^2^/g)	Micropore Volume (cm^3^/g)	Pore Volume (cm^3^/g)
ZIF-8	1551	0.55	0.58
ZIF-8-m	942	0.35	0.37
ZIF-67	1833	0.70	0.72
ZIF-67-m	914	0.36	0.40

## Data Availability

Not applicable.

## Appendix A

Epichlorohydrin. ^1^H-NMR (300 MHz, CDCl_3_) δ = 3.67-3.54 (m, 1H), 3.50-3.44 (m, 1H), 3.26-3.15 (m, 1H), 2.85 (dd, *J* = 4.8, 3.9 Hz, 1H), 2.65 (dd, *J* =4.8, 2.5 Hz, 1H).

4-(Chloromethyl)-1,3-dioxolan-2-one. ^1^H-NMR (300 MHz, CDCl_3_) δ = 5.00-4.94 (m, 1H), 4.57 (dd, *J* = 8.9, 8.3 Hz, 1H), 4.37 (dd, *J* = 8.9, 5.7 Hz, 1H), 3.82-3.77 (m, 1H), 3.72-3.65 (m, 1H).

## References

[1] Pescarmona P.P. (2021). Cyclic carbonates synthesised from CO_2_: Applications, challenges and recent research trends. Curr. Opin. Green Sustain. Chem..

[2] Kamphuis A.J., Picchioni F., Pescarmona P.P. (2019). CO_2_-fixation into cyclic and polymeric carbonates: Principles and applications. Green Chem..

[3] Maltby K.A., Hutchby M., Plucinski P., Davidson M.G., Hintermair U. (2020). Selective Catalytic Synthesis of 1,2- and 8,9-Cyclic Limonene Carbonates as Versatile Building Blocks for Novel Hydroxyurethanes. Chem.-A Eur. J..

[4] Liu J., Li Y., Liu H., He D. (2018). Transformation of CO_2_ and glycerol to glycerol carbonate over CeO_2_–ZrO_2_ solid solution—Effect of Zr doping. Biomass Bioenergy.

[5] Razali N., McGregor J. (2021). Improving product yield in the direct carboxylation of glycerol with CO_2_ through the tailored selection of dehydrating agents. Catalysts..

[6] Rozulan N., Halim S.A., Razali N., Lam S.S. (2022). A review on direct carboxylation of glycerol waste to glycerol carbonate and its applications. Biomass Convers. Biorefinery.

[7] Duan C., Yu Y., Hu H. (2022). Recent progress on synthesis of ZIF-67-based materials and their application to heterogeneous catalysis. Green Energy Environ..

[8] Xiang W., Sun Z., Wu Y., He L., Liu C. (2020). Enhanced cycloaddition of CO_2_ to epichlorohydrin over zeolitic imidazolate frameworks with mixed linkers under solventless and co-catalyst-free condition. Catal. Today.

[9] Zhou K., Mousavi B., Luo Z., Phatanasri S., Chaemchuen S., Verpoort F. (2017). Characterization and properties of Zn/Co zeolitic imidazolate frameworks vs. ZIF-8 and ZIF-67. J. Mater. Chem. A..

[10] Kuruppathparambil R.R., Jose T., Babu R., Hwang G.Y., Kathalikkattil A.C., Kim D.W., Park D.W. (2016). A room temperature synthesizable and environmental friendly heterogeneous ZIF-67 catalyst for the solvent less and co-catalyst free synthesis of cyclic carbonates. Appl. Catal. B Environ..

[11] Caló V., Nacci A., Monopoli A., Fanizzi A. (2002). Cyclic carbonate formation from carbon dioxide and oxiranes in tetrabutylammonium halides as solvents and catalysts. Org. Lett..

[12] Mazo P., Rios L. (2013). Carbonation of epoxidized soybean oil improved by the addition of water. J. Am. Oil Chem. Soc..

[13] Anderson C.E., Vagin S.I., Xia W., Jin H., Rieger B. (2012). Cobaltoporphyrin-catalyzed CO_2_/epoxide copolymerization: Selectivity control by molecular design. Macromolecules.

[14] Sugimoto H., Kuroda K. (2008). The cobalt porphyrin–Lewis base system: A highly selective catalyst for alternating copolymerization of CO_2_ and epoxide under mild conditions. Macromolecules.

[15] Udayakumar S., Lee M.K., Shim H.L., Park S.W., Park D.W. (2009). Imidazolium derivatives functionalized MCM-41 for catalytic conversion of carbon dioxide to cyclic carbonate. Catal. Commun..

[16] Barbarini A., Maggi R., Mazzacani A., Mori G., Sartori G., Sartorio R. (2003). Cycloaddition of CO_2_ to epoxides over both homogeneous and silica-supported guanidine catalysts. Tetrahedron Lett..

[17] Aprile C., Giacalone F., Agrigento P., Liotta L.F., Martens J.A., Pescarmona P.P., Gruttadauria M. (2011). Multilayered supported ionic liquids as catalysts for chemical fixation of carbon dioxide: A high-throughput study in supercritical conditions. ChemSusChe.

[18] Cheng W., Chen X., Sun J., Wang J., Zhang S. (2013). SBA-15 supported triazolium-based ionic liquids as highly efficient and recyclable catalysts for fixation of CO_2_ with epoxides. Catal. Today.

[19] Taherimehr M., Van de Voorde B., Wee L.H., Martens J.A., De Vos D.E., Pescarmona P.P. (2017). Strategies for Enhancing the Catalytic Performance of Metal–Organic Frameworks in the Fixation of CO_2_ into Cyclic Carbonates. ChemSusChem.

[20] Zalomaeva O.V., Maksimchuk N.V., Chibiryaev A.M., Kovalenko K.A., Fedin V.P., Balzhinimaev B.S. (2013). Synthesis of cyclic carbonates from epoxides or olefins and CO_2_ catalyzed by metal-organic frameworks and quaternary ammonium salts. J. Energy Chem..

[21] Ramos-Fernández E.V., Serrano-Ruiz J.C., Sepúlveda-Escribano A., Narciso J., Ferrando-Soria J., Pardo E., Reina T.R., Odriozola J.A. (2020). CHAPTER 9 Metal Organic Frameworks: From Material Chemistry to Catalytic Applications. Heterogeneous Catalysis for Energy Applications.

[22] Delgado-Marín J.J., Izan D.P., Molina-Sabio M., Ramos-Fernandez E.V., Narciso J. (2022). New Generation of MOF-Monoliths Based on Metal Foams. Molecules.

[23] Ronda-Lloret M., Pellicer-Carreño I., Grau-Atienza A., Boada R., Diaz-Moreno S., Narciso-Romero J., Serrano-Ruiz J.C., Sepúlveda-Escribano A., Ramos-Fernandez E.V. (2021). Mixed-Valence Ce/Zr Metal-Organic Frameworks: Controlling the Oxidation State of Cerium in One-Pot Synthesis Approach. Adv. Funct. Mater..

[24] Ramos-Fernandez E.V., Redondo-Murcia A., Grau-Atienza A., Sepúlveda-Escribano A., Narciso J. (2020). Clean production of Zeolitic Imidazolate Framework 8 using Zamak residues as metal precursor and substrate. J. Clean. Prod..

[25] Beyzavi M.H., Stephenson C.J., Liu Y., Karagiaridi O., Hupp J.T., Farha O.K. (2015). Metal-organic framework-based catalysts: Chemical fixation of CO_2_ with epoxides leading to cyclic organic carbonates. Front. Energy Res..

[26] Ouellette W., Hudson B.S., Zubieta J. (2007). Hydrothermal and Structural Chemistry of the Zinc(II)- and Cadmium(II)-1,2,4-Triazolate Systems. Inorg. Chem..

[27] Oullette W., Prosvirin A.V., Valeich J., Dunbar K.R., Zubieta J. (2007). Hydrothermal Synthesis, Structural Chemistry, and Magnetic Properties of Materials of the M^II^/Triazolate/Anion Family, Where M^II^ = Mn, Fe, and Ni. Inorg. Chem..

[28] Bondarenko G.N., Dvurechenskaya E.G., Ganina O.G., Alonso F., Beletskaya I.P. (2019). Solvent-free Synthesis of Cyclic Carbonates from CO_2_ and Epoxides Catalyzed by Reusable Alumina-supported Zinc Dichloride. Appl. Catal. B: Environ..

[29] Kuruppathparambil R.R., Babu R., Jeong H.M., Hwang G.Y., Jeong G.S., Kim M.I., Kim D.W., Park D.W. (2016). A Solid SolutionZeolitic Imidazolate Framework as a Room Temperature Efficient Catalyst for the Chemical Fixation of CO_2_. Green Chem..

[30] Mousavi B., Chaemchuen S., Moosavi B., Luo Z., Gholampour N., Verpoort F. (2016). Zeolitic Imidazole Framework-67 as an Efficient Heterogeneous Catalyst for the Conversion of CO_2_ to Cyclic Carbonates. New J. Chem..

[31] Chizallet C., Lazare S., Bazer-Bachi D., Bonnier F., Lecocq V., Soyer E., Quoineaud A.A., Bats N. (2010). Catalysis of Transesterification by a Nonfunctionalized Metal−Organic Framework: Acido-Basicity at the External Surface of ZIF-8 Probed by FTIR and ab Initio Calculations. J. Am. Chem. Soc..

[32] Blackman A. (1993). Reactions of Coordinated ligands. Adv. Heterocycl. Chem..

[33] Cox J.R., Woodcock S., Hillier I.H., Vincent M.A. (1990). Tautomerism of 1,2,3- and 1,2,4-Triazole in the Gas Phase and In Aqueous Solution: A Combined ab Initio Quantum Mechanics and Free Energy Perturbation Study. J. Phys. Chem..

[34] Lunazi L., Parisi F., Macciantelli D. (1984). Conformational studies by dynamic nuclear magnetic resonance spectroscopy. Part 27. Kinetics and mechanism of annular tautomerism in isomeric triazoles. J. Chem. Soc. Perkin Trans. 2.

[35] Garrat P.J., Katrizky A.R., Rees C.W., Scriven E.F.V., Storr R.C. (1996). Comprehensive Heterocyclic Chemistry II.

[36] Narciso J., Ramos-Fernandez E.V., Delgado-Marín J.J., Affolter C.W., Olsbye U., Redekop E.A. (2021). New route for the synthesis of Co-MOF from metal substrates. Microporous Mesoporous Mater..

[37] Villalgordo-Hernández D., Grau-Atienza A., García-Marín A.A., Ramos-Fernández E.V., Narciso J. (2022). Manufacture of Carbon Materials with High Nitrogen Content. Materials.

